# Faster Fertilization and Cleavage Kinetics Reflect Competence to Achieve a Live Birth: Data from Single-Embryo Transfer Cycles

**DOI:** 10.1155/2022/8501362

**Published:** 2022-07-15

**Authors:** Yongle Yang, Xiyuan Dong, Jian Bai, Lei Jin, Bo Huang

**Affiliations:** Reproductive Medicine Center, Tongji Hospital, Tongji Medical College, Huazhong University of Science and Technology, Wuhan, China

## Abstract

The aim of this study was to assess the relationship between early developmental kinetics and the competence to result in a live birth as well as the impact of maternal age and the number of retrieved oocytes. This retrospective cohort study included 3,021 single-embryo transfer cycles and assessed live birth outcomes paired with morphokinetic data; 1,412 transfers resulted in live births (LB), and 1,609 did not (NLB). Early morphokinetic parameters between LB and NLB embryos were compared from patients stratified into four age groups (20-25, 26-30, 31-36, and ≥37 years) and between embryos in the same competence groups within the age groups. Early morphokinetic parameters were also compared between LB and NLB embryos from patients stratified into four groups based on the number of oocytes harvested (≤7, 8-14, 15-21, and ≥22). The association between morphokinetic parameters and LB was tested using univariate and multivariate analyses. This study indicated that embryos resulting in LB generally exhibit faster developmental dynamic parameters than embryos that do not. However, this difference decreased in the younger (20-25 years) and older (≥37 years) age groups. In addition, when the number of harvested oocytes was low (≤7) or high (≥22), this difference was less obvious. The morphokinetic parameters of embryonic cleavage are an effective reference value for embryo selection strategies aimed at increasing live birth rates, especially for patients aged 26–36 years, with 8–21 harvested oocytes.

## 1. Introduction

The outcomes of in vitro fertilization (IVF) critically depend on selecting the best embryo to be transferred, a choice that is predominantly based on static morphologic criteria and the medical history of the patient. Over the past decade, time-lapse technology has been applied widely in assisted reproductive technology (ART). Time-lapse imaging (TLI) has allowed for more detailed assessment of embryo morphological dynamics, although its ability to improve the clinical success of IVF has not been confirmed [1]. Integrating genetic, metabolic, and morphological data using artificial intelligence will greatly improve the accuracy of embryo selection [2]. It is essential to elucidate the potential utility and limitations of morphokinetic information to optimize the performance of time-lap-based strategies and develop comprehensive methods for embryo selection. TLI has been used to evaluate embryonic developmental dynamics and morphological parameters and to determine their relationships with laboratory and clinical results [3–5].

Numerous studies show that faster developmental kinetics are related to higher cell numbers [6, 7], better blastocyst formation [8–11], and higher implantation [12, 13] and pregnancy rates [4, 14]. Although these studies provide a theoretical basis for developing TLI-based embryo selection algorithms aimed at improving implantation and pregnancy rates [15–17], investigation of the impact on live birth rates needs to be performed [18].

At present, there are few reports on the relationship between embryo dynamics and live births. This presents a gap in the application of TLI technology in clinical practice [18]. A recent report found that blastocyst morphological dynamics, specifically the beginning of development and overall duration, can be used to predict live birth and have greater discriminating power than conventional blastocyst morphology [19]. However, the prediction of live birth using embryo morphological dynamics may not be applicable to the entire developmental stage of the embryo. Another study showed no correlation between fertilization or cleavage parameters and live births; however, this result may be related to the limited sample size of this study [20]. Our early data suggest that the sex of live births is related to embryonic developmental dynamics [21].

The accuracy of embryo selection algorithms based on TLI has been questioned [18, 22]. This may be because the data were obtained from different centers and differences in morphokinetic parameters are largely affected by varying culture conditions, the conditions of patients, and operators' practices from different centers [22, 23]. Studies report that the age of the parturient is related to the morphological and developmental dynamics of the embryo, but the effects of age on the dynamic parameters of the embryo have not been completely resolved [24, 25]. Several early studies explored the relationship between age and cleavage morphological dynamics [26–28]. One study demonstrated that compared with older (30-40 years) patients, younger (20-30 years) patients had an earlier tPNf (time of pronuclear fading), t2 (time when two separate and distinct cells were identified), t3 (time at which a 3-blastomere embryo was identified), and t4 (time when a 4-blastomere embryo was identified) [28]. However, these early studies failed to describe the effects of embryo quality and maternal age on morphological dynamics. Slower embryo morphological dynamic parameters are to be expected because of the higher proportion of inferior embryos in older parturients [29]. Therefore, the relationship between patient age and embryo morphological dynamics requires further clarification.

Previous studies have shown that the number of oocytes obtained is related to embryo quality [30], maternal age, and live birth rate [31]. Therefore, we included the following aspects in our study: maternal age, number of oocytes retrieved, and live births. In the aim to clarify the use of TLI in observing the kinetic parameters of embryos, as well as to identify the potential clinical value, we proposed the following hypotheses: (1) embryos capable of live births have faster developmental kinetic parameters, (2) maternal age affects the kinetic parameters of early embryo development and the embryo's ability to result in a live birth, and (3) the number of oocytes retrieved affects the kinetic parameters of embryo development and the ability to result in a live birth.

## 2. Methods

### 2.1. Patients and Experimental Design

Due to the observational nature of retrospective studies, it should not be assumed that there is a causal relationship between live births and maternal age or the number of oocytes retrieved; controlled experiments need to be carefully designed before providing clinical recommendations. We retrospectively studied the ART cycles at our center from January 2018 to May 2019. The main indicators were live births and nonlive births, maternal age (20-25, 26-30, 31-36, and ≥37 years), and ovarian response (≤7, 8-14, 15-21, and ≥22 oocytes). This study included 3,021 patients and was conducted at the Reproductive Medicine Center of Tongji Hospital, Tongji Medical College, Huazhong University of Science and Technology. All the procedures and protocols were approved by the ethics committee of Reproductive Medicine Center, Tongji Hospital, Tongji College of Medicine, Huazhong University of Science and Technology. After the patients underwent IVF/intracytoplasmic sperm injection (ICSI) treatment, we used morphological parameters to select the most suitable embryos for transfer on the third day. It has been established that there is a significant difference in ectopic pregnancy rates between fresh embryos with day 3 embryo transfer and frozen embryos with day 3 embryo transfer [32]. To eliminate the influence of this difference, we collected all our data from the fresh embryo transfer cycle. Studies show that there is no difference in the live birth rate between conventional and early rescue ICSI [33]. If there is early rescue ICSI in a cycle, the developmental kinetic parameters have no reference value; therefore, our data excluded the early rescue ICSI cycle.

We matched all embryos used for transfer with their dynamic parameters and studied the relationship between the morphological dynamics and the mother's age and live birth as well as the relationship between the morphological dynamics and the number of oocytes retrieved and live birth. We compared developmental kinetic parameters between these groups to determine the relationship between faster embryo kinetic parameters and the ability to result in live births. To test the effect of maternal age on embryonic development dynamics, we divided the patients into four age groups (20-25, 26-30, 31-36, and ≥37 years). We compared the developmental dynamic parameters of the four groups to LB (live birth achieved, *n* = 1, 412) and NLB (live birth not achieved, *n* = 1, 609; nonlive birth included biochemical pregnancy and miscarriage) embryos, respectively. To test the relationship between the number of retrieved oocytes and embryonic development kinetics and live birth, we divided the patients into four groups (≤7, 8-14, 15-21, and ≥22 oocytes) according to the number of oocytes retrieved. We compared the developmental kinetic parameters of the different groups of embryos (Table 1).

### 2.2. Embryo Culture

Embryos were cultured according to conventional methods. After the oocytes were collected, the cumulus-oocyte complex was cultured in fertilization medium (G-IVF; Vitrolife). A discontinuous gradient solution (45% and 90%; SpermGrad, Vitrolife) was used to wash the spermatozoa, and the obtained sperm pellet was placed at the bottom of the fertilization medium. In the IVF group, the optimized upper sperm suspension and cumulus-oocyte mixture were combined and cultured in the fertilization medium for short-term fertilization. After 3 hours, the granular cells were removed by mechanical action using an in vitro fertilization micromanipulation tube (Cook Vandergrift Inc.). Embryo culture was performed using an integrated embryo culture time lapse microscopy system (Embryo Scope; Vitrolife), where images were taken every ten minutes in seven different focal planes. In the ICSI group, the cumulus-oocyte complex was placed in contact with human recombinant hyaluronidase (80 IU/mL) for a short time, and the granulosa cells were peeled off by mechanical action. After ICSI, oocytes were transferred to a prepared TL dish and cultured in the time lapse microscopy system. All the embryos were cultured in a culture device under the same conditions.

### 2.3. Morphokinetic Parameters

All embryos in this study were cultured in a TLI system, and related developmental events were identified using EmbryoViewer (Vitrolife) after fertilization. The morphokinetic parameters evaluated in this study were: tPNf, t2 (time to appearance of two blastomeres), t3 (time to appearance of three blastomeres), t4 (time to appearance of four blastomeres), t5 (time to appearance of five blastomeres), and t8 (time to appearance of eight blastomeres). The morphokinetic parameters, tPNf, t2, t3, t4, and t5, were used in the annotation of all embryos; however, due to the limitation of the transfer time, t8 could not annotate all embryos. In addition, to avoid the interference of morphokinetic parameters due to different fertilization times in IVF and ICSI, we also observed the time when the first pronucleus appeared in all embryos. We recorded the times of tPNf, t2, t3, t4, t5, and t8 along with the time when the first pronucleus appeared as a starting point to standardize all parameters in different groups. The annotation of the morphokinetic parameters was completed by ten senior embryologists with more than five years of experience in embryo laboratory procedures.

### 2.4. Embryo Scoring, Selection, and Transfer

According to the Istanbul consensus [34], we used digital images to evaluate the morphology of embryos on the second and third day. The choice of embryo transfer was based entirely on static morphological criteria. Embryo transfer strategies are generally based on the mother's age and the medical history of both the mother and father as well as the number of embryos and embryo quality. Fresh embryos were transferred on the third day, and the remaining embryos were used to culture blastocysts, frozen, and preserved for subsequent use. *β*-HCG (*β*-Human chorionic gonadotropin) was detected 12 days after embryo transfer, and clinical pregnancy was diagnosed by ultrasound examination in the seventh week after embryo transfer. All pregnancies were followed, and the primary outcome was the delivery of one or more live infants; this was confirmed through the review of medical records. The live birth rate was calculated using the fresh embryo transfer cycle and the corresponding live birth end.

### 2.5. Statistical Analysis

The mean values and standard deviations were used to describe the continuous characteristics; the chi-square test was used to compare the distribution of infertility causes between live births and nonlive births. Univariate and multivariate logistic analyses were used to analyze embryo morphokinetic parameters, maternal variables, and the relationship between the number of oocytes harvested and live births. Origin software (Origin 9.0, Origin Lab, Northampton, USA) was used, and *p* < 0.05 was considered statistically significant.

## 3. Results

### 3.1. Patient Characteristics and General Outcomes

This study included single or more IVF/ICSI cycles performed at our center between January 2018 and May 2019. We studied the embryos used for single-embryo transfer on the third day in all the patients. The overall live birth rate was 42.54%. All embryos used for transfer were matched with their kinetic parameters and used to evaluate their relationship with live birth, maternal age, and number of oocytes harvested. The basic clinical characteristics of the included population are shown in Table 2. Patients who had a live birth were younger and yielded a larger number of oocytes but presented a similar BMI compared to those who provided only NLB embryos.

LB: live birth achieved; NLB: live birth not achieved; BMI: Body Mass Index.

### 3.2. Effects of Embryo Competence and Maternal Age on Early Morphokinetics

**Figure 1:**
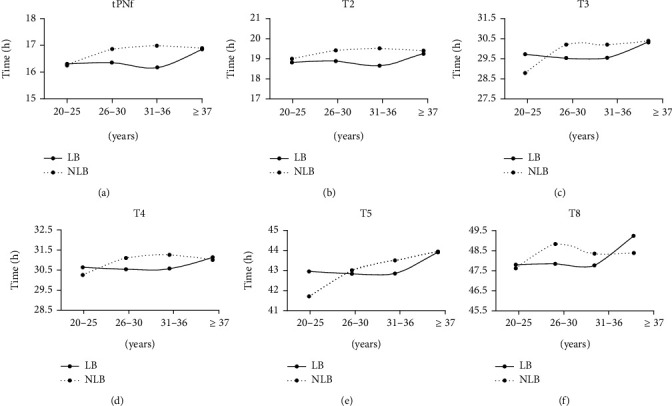
Line graphs of various morphokinetic parameters for different age groups. (a) Morphokinetic parameters of tPNf. (b) Morphokinetic parameters of t2. (c) Morphokinetic parameters of t3. (d) Morphokinetic parameters of t4. (e) Morphokinetic parameters of t5. (f) Morphokinetic parameters of t8.

As shown in Table 1 and Figure 1, when all the samples were analyzed together, the kinetic endpoints (tPNf, t2, t3, t4, t5, and t8) of all embryos that resulted in an LB were reached earlier than those for NLB embryos. In addition, among the embryos provided by patients between the ages of 26 and 36 years, the kinetic endpoints of embryos that resulted in a LB were reached earlier than those of NLB (Table 3). There was no significant difference in the embryo dynamic endpoints between younger patients (20-25 years) and older patients (≥37 years).

### 3.3. Effects of Embryo Competence and the Number of Oocytes Retrieved from Early Morphokinetics

**Figure 2:**
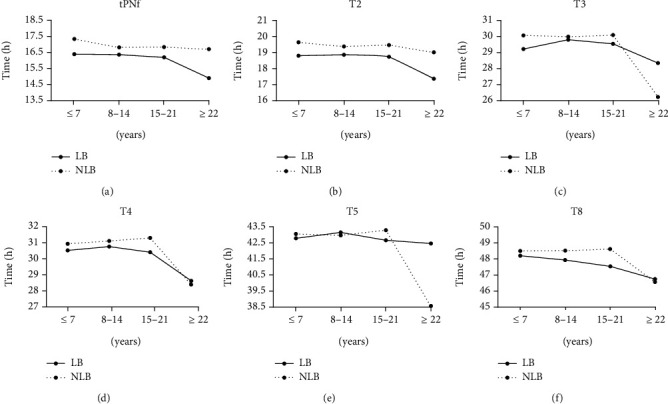
Morphokinetic parameters of embryos provided by patients with different numbers of retrieved oocytes. (a) Morphokinetic parameters of tPNf. (b) Morphokinetic parameters of t2. (c) Morphokinetic parameters of t3. (d) Morphokinetic parameters of t4. (e) Morphokinetic parameters of t5. (f) Morphokinetic parameters of t8.

When we grouped the patients according to the number of oocytes obtained, we observed an interesting phenomenon. As shown in Table 4 and Figure 2, compared with NLB, embryos resulting in LB had an earlier dynamic endpoint (tPNf, t2, t3, t4, t5, and t8). However, the embryos provided by patients with a larger number of oocytes were different. When the number of oocytes obtained was greater than 22, the dynamic endpoint parameters of the embryos resulting in LB and NLB exhibited the opposite phenomenon. In other words, NLB embryos showed an earlier kinetic endpoint (tPNf, t2, t3, t4, t5, and t8) in patients with higher oocyte numbers.

hrs.: hours; LB: live birth achieved; NLB: live birth not achieved; tPNf: time of pronuclear fading; ^a^*P* < 0.05; ^b^0.01 < *P* < 0.001; ^c^*P* < 0.001; ^NS^*P* > 0.05.

## 4. Discussion

The current application of TLI in embryo selection remains controversial in terms of whether it increases live birth rate. However, TLI is still widely used in embryo laboratories. Our data indicated that the kinetics of embryo fertilization and cleavage are related to the ability to result in live births and embryos that provide LB tend to show earlier kinetic parameters. This phenomenon was related to the age of the parturient (20-36 years). When the age of the parturient was >37 years, this phenomenon was not observed. Faster tPNf and t2 are believed to be related to embryo morphology on the third day [7]; however, this is not always the case. There are reports that early embryo morphokinetics are better predictors of post-ICSI live births than embryo morphology [6]. Earlier t3, t4, and t5 are related to higher implantation rates, whereas t4 reflects the quality of blastocysts [12, 35].

In addition, a shorter interval between t5 and t8 is related to blastocyst formation [7, 36]. These reports suggest that an earlier kinetic endpoint is positively correlated with the morphological quality of the embryo. However, among embryos with appropriate morphology, a considerable proportion (20.1%) showed poor kinetic parameters, accompanied by a severely reduced live birth rate (*P* < 0.001). Compared to traditional morphology, early morphological dynamics can predict live births more accurately [6]. This indicates that early embryonic morphological dynamics can be used as an important parameter for embryo selection and transfer.

It is a popular belief that higher maternal age reduces IVF success rate [37, 38]. This may be one of the clinical factors relating to inconsistent results based on German TLI strategies [23]. The design of experiments that investigate the influence of maternal age on embryo morphological dynamics is complicated by different age thresholds. For example, one German study used 38 to 42 years as the critical value and found that the age of the mother did not affect cleavage kinetics [26, 27]. Another study used 20 to 30 years and 30 to 40 years as the critical values and showed that the mother's age delayed tPNf and cleavage kinetics to t4 [28].

We demonstrated that the maternal age variable affects the developmental dynamics of embryos and their ability to provide live births. Another interesting phenomenon is that there are significant differences in fertilization and cleavage between LB and NLB embryos when the mother is 26–36 years old; the kinetic parameters of the embryos resulting in LB were slower than those of the embryos with NLB. When the mother was older than 37 years or between 20 and 25 years, there was no significant difference in various kinetic parameters (Figure 1). This also reflects the impact of the mother's age on live births and the difference in the dynamics of LB and NLB embryos. Interestingly, our data suggests that age has an inconsistent effect on embryo dynamics and the ability to provide live births. This is reflected in the 20-25-year-old age group [25]. This difference may be related to the age used as a cutoff value or the number of embryos transferred. Our research is limited by its retrospective nature, the use of data generated by a single reproductive center, the unevenness of the age groups in our database, and the differences between operators, all of which may have affected our results.

We also observed that LB embryos tended to have earlier endpoints of early kinetic parameters, and this phenomenon was also related to the number of oocytes harvested. When the number of oocytes obtained was less than 21, the early kinetic parameters of LB embryos reached the endpoint earlier than those of NLB embryos. There was no difference between LB and NLB when the number of oocytes obtained was greater than 22. Existing research on the number of oocytes harvested in ART show inconsistent results regarding the relationship between the number of oocytes obtained and the pregnancy rate. For example, studies show that pregnancy rate increases with the number of oocytes retrieved [39], and the best pregnancy rate can be obtained when the number of oocytes retrieved is 10-15 [40] or 5-15 [41]. However, these studies were based on small sample sizes from a single center, which limits their generality. There have been studies on the relationship between the number of oocytes obtained, the age of the patient, and live births, and it was found that the optimal number of oocytes obtained in a fresh IVF cycle (approximately 15) can also maximize the success rate while reducing the risk of ovarian hyperstimulation syndrome (OHSS) [42]. Our study is the first to investigate the number of oocytes obtained and developmental dynamics of LB and NLB embryos.

## 5. Conclusions

The goal of every ART cycle is to result in a live birth; therefore, it is very important for patients and reproduction practitioners to understand the factors predicting the success of IVF/ICSI. These factors help provide consultation for patients trying to decide which treatment plan to adopt. Our research based on the patient's age and the number of oocytes obtained provided details on the early developmental dynamics of LB and NLB embryos and identified parameters that influence these developmental dynamics. These findings provide additional reference information for optimal embryo selection.

## Figures and Tables

**Table 1:** Fertilization and cleavage morphokinetic parameters of all embryos produced.

Parameter	Total (*N* = 3021)	LB (*N* = 1412)	NLB (*N* = 1609)	*P* value
tPNf (hrs.)	16.59 ± 2.48	16.31 ± 2.25	16.84 ± 2.64	*P* < 0.001
t2 (hrs.)	19.14 ± 2.59	18.82 ± 2.24	19.41 ± 2.84	*P* < 0.001
t3 (hrs.)	29.86 ± 3.53	29.72 ± 3.20	29.98 ± 3.79	*P* = 0.17
t4 (hrs.)	30.89 ± 3.29	30.65 ± 3.02	31.09 ± 3.49	*P* = 0.011
t5 (hrs.)	43.05 ± 5.07	42.97 ± 4.92	43.12 ± 5.21	*P* = 0.58
t8 (hrs.)	48.17 ± 4.92	47.81 ± 4.74	48.5 ± 5.56	*P* = 0.010

hrs.: hours; LB: live birth achieved; NLB: live birth not achieved; tPNf: time of pronuclear fading.

**Table 2:** Baseline clinical characteristics for all patients, patients providing embryos that were transferred but did not result in a live birth, and patients who achieved a live birth.

Characteristic	Total (*N* = 3021)	LB (*N* = 1412)	NLB (*N* = 1609)	*P* value
Maternal age (years)	30.39 ± 15.7	29.77 ± 12.31	30.89 ± 18.07	*P* < 0.001
Maternal BMI (kg/m^2^)	22.04 ± 9.19	21.96 ± 9.12	22.05 ± 9.235	*P* = 0.41
Number of oocytes retrieved	12.91 ± 17.94	13.07 ± 17.44	12.77 ± 18.42	*P* = 0.053

**Table 3:** Morphokinetic parameters of all embryos produced in different maternal age groups.

20-25 (y)			26-30 (y)		31-36 (y)		≥37 (y)	
Parameter	LB	NLB	LB	NLB	LB	NLB	LB	NLB
tPNf (hrs.)	16.30 ± 2.25^NS^ (*n* = 669)	16.25 ± 2.54^NS^ (*n* = 668)	16.35 ± 2.20^a^ (*n* = 424)	16.85 ± 2.72^a^ (*n* = 476)	16.16 ± 2.22^c^ (*n* = 249)	16.97 ± 2.54^c^ (*n* = 382)	16.84 ± 2.19^NS^ (*n* = 70)	16.89 ± 2.68^NS^ (*n* = 83)
t2 (hrs.)	18.82 ± 2.24^NS^ (*n* = 669)	19.00 ± 3.17^NS^ (*n* = 668)	18.88 ± 2.22^b^ (*n* = 424)	19.42 ± 2.94^b^ (*n* = 476)	18.65 ± 2.18^c^ (*n* = 249)	19.52 ± 2.68^c^ (*n* = 382)	19.26 ± 2.16^NS^ (*n* = 70)	19.41 ± 2.62^NS^ (*n* = 83)
t3 (hrs.)	29.73 ± 3.21^a^ (*n* = 669)	28.79 ± 4.10^a^ (*n* = 668)	29.55 ± 3.16^a^ (*n* = 424)	30.20 ± 3.93^a^ (*n* = 476)	29.55 ± 3.23^a^ (*n* = 249)	30.20 ± 3.49^a^ (*n* = 382)	30.34 ± 3.24^NS^ (*n* = 70)	30.40 ± 3.73^NS^ (*n* = 83)
t4 (hrs.)	30.66 ± 3.02^NS^ (*n* = 669)	30.30 ± 4.05^NS^ (*n* = 668)	30.57 ± 2.96^a^ (*n* = 424)	31.13 ± 3.66^a^ (*n* = 476)	30.60 ± 3.04^a^ (*n* = 249)	31.28 ± 3.12^a^ (*n* = 382)	31.15 ± 2.78^NS^ (*n* = 70)	31.05 ± 3.41^NS^ (*n* = 83)
t5 (hrs.)	42.96 ± 4.92^NS^ (*n* = 669)	41.72 ± 5.42^NS^ (*n* = 668)	42.87 ± 4.96^NS^ (*n* = 424)	43.01 ± 5.04^NS^ (*n* = 476)	42.87 ± 5.01^NS^ (*n* = 249)	43.52 ± 5.58^NS^ (*n* = 382)	43.92 ± 4.37^NS^ (*n* = 70)	43.95 ± 5.56^NS^ (*n* = 83)
t8 (hrs.)	47.80 ± 4.74^NS^ (*n* = 644)	47.65 ± 5.33^NS^ (*n* = 665)	47.84 ± 4.53^a^ (*n* = 410)	48.83 ± 5.19^a^ (*n* = 460)	47.79 ± 4.99^NS^ (*n* = 235)	48.34 ± 4.86^NS^ (*n* = 369)	49.24 ± 4.45^NS^ (*n* = 68)	48.39 ± 4.67^NS^ (*n* = 77)

hrs.: hours; LB: live birth achieved; NLB: live birth not achieved; tPNf: time of pronuclear fading; ^a^*P* < 0.05; ^b^0.01 < *P* < 0.001; ^c^*P* < 0.001; ^NS^*P* > 0.05.

**Table 4:** Morphokinetic parameters of all embryos produced in different number of oocytes retrieved.

Oocytes	≤7		8-14		15-21		≥22	
Parameter	LB	NLB	LB	NLB	LB	NLB	LB	NLB
tPNf (hrs.)	16.39 ± 2.43^NS^ (*n* = 120)	17.35 ± 2.39^NS^ (*n* = 176)	16.37 ± 2.21^b^ (*n* = 798)	16.83 ± 2.68^b^ (*n* = 900)	16.20 ± 2.29^b^ (*n* = 447)	16.83 ± 2.56^b^ (*n* = 481)	14.88 ± 1.45^NS^ (*n* = 47)	16.72 ± 2.48^NS^ (*n* = 52)
t2 (hrs.)	18.80 ± 2.45^NS^ (*n* = 120)	19.65 ± 2.49^NS^ (*n* = 176)	18.87 ± 2.19^b^ (*n* = 798)	19.39 ± 2.79^b^ (*n* = 900)	18.76 ± 2.31^b^ (*n* = 447)	19.47 ± 2.99^b^ (*n* = 481)	17.40 ± 1.74^NS^ (*n* = 47)	19.03 ± 2.40^NS^ (*n* = 52)
t3 (hrs.)	29.23 ± 4.41^NS^ (*n* = 120)	30.06 ± 3.89^NS^ (*n* = 176)	29.85 ± 3.06^NS^ (*n* = 798)	29.98 ± 3.77^NS^ (*n* = 900)	29.55 ± 3.31^NS^ (*n* = 447)	30.10 ± 3.74^NS^ (*n* = 481)	28.33 ± 2.41^NS^ (*n* = 47)	26.22 ± 3.23^NS^ (*n* = 52)
t4 (hrs.)	30.53 ± 3.27^NS^ (*n* = 120)	30.93 ± 3.22^NS^ (*n* = 176)	30.79 ± 2.85^NS^ (*n* = 798)	31.09 ± 3.48^NS^ (*n* = 900)	30.42 ± 3.31^b^ (*n* = 447)	31.28 ± 3.48^b^ (*n* = 481)	28.65 ± 2.21^NS^ (*n* = 47)	28.4 ± 2.54^NS^ (*n* = 52)
t5 (hrs.)	42.79 ± 6.75^NS^ (*n* = 120)	43.05 ± 5.92^NS^ (*n* = 176)	43.13 ± 4.76^NS^ (*n* = 798)	42.99 ± 5.11^NS^ (*n* = 900)	42.66 ± 4.96^NS^ (*n* = 447)	43.30 ± 5.61^NS^ (*n* = 481)	42.45 ± 3.87^NS^ (*n* = 47)	38.58 ± 3.57^NS^ (*n* = 52)
t8 (hrs.)	48.20 ± 4.94^NS^ (*n* = 107)	48.48 ± 4.96^NS^ (*n* = 168)	47.92 ± 4.75^a^ (*n* = 774)	48.50 ± 5.20^a^ (*n* = 884)	47.52 ± 4.63^a^ (*n* = 432)	48.60 ± 4.73^a^ (*n* = 472)	46.75 ± 5.49^NS^ (*n* = 44)	46.53 ± 2.15^NS^ (*n* = 47)

## Data Availability

All data used to support the findings of this study are included within the article.
